# C-reactive protein as a potential biomarker for disease progression in dengue: a multi-country observational study

**DOI:** 10.1186/s12916-020-1496-1

**Published:** 2020-02-17

**Authors:** Nguyen Lam Vuong, Huynh Thi Le Duyen, Phung Khanh Lam, Dong Thi Hoai Tam, Nguyen Van Vinh Chau, Nguyen Van Kinh, Ngoun Chanpheaktra, Lucy Chai See Lum, Ernesto Pleités, Nick Keith Jones, Cameron Paul Simmons, Kerstin Rosenberger, Thomas Jaenisch, Christine Halleux, Piero Luigi Olliaro, Bridget Wills, Sophie Yacoub

**Affiliations:** 1 Oxford University Clinical Research Unit, Wellcome Trust Asia Programme, Ho Chi Minh City, Vietnam; 2 University of Medicine and Pharmacy at Ho Chi Minh City, Ho Chi Minh City, Vietnam; 3 Hospital of Tropical Diseases, Ho Chi Minh City, Vietnam; 4 National Hospital for Tropical Diseases (NHTD), Hanoi, Vietnam; 5 Angkor Hospital for Children, Siem Reap, Cambodia; 6 University of Malaya Medical Centre, Kuala Lumpur, Malaysia; 7 Hospital Nacional de Niños Benjamin Bloom, San Salvador, El Salvador; 8 University of Cambridge, Cambridge, UK; 9 Institute of Vector-Borne Disease, Monash University, Melbourne, Australia; 10 Section of Clinical Tropical Medicine, Department of Infectious Diseases, Heidelberg University Hospital, Heidelberg, Germany; 11 UNICEF/UNDP/World Bank/WHO Special Programme for Research and Training in Tropical Diseases, World Health Organization, Geneva, Switzerland; 12 Centre for Tropical Medicine and Global Health, Nuffield Department of Medicine, University of Oxford, Oxford, UK

**Keywords:** Dengue, Biomarker, C-reactive protein, Prognosis, Other febrile illness

## Abstract

**Background:**

Dengue infection can cause a wide spectrum of clinical outcomes. The severe clinical manifestations occur sufficiently late in the disease course, during day 4–6 of illness, to allow a window of opportunity for risk stratification. Markers of inflammation may be useful biomarkers. We investigated the value of C-reactive protein (CRP) measured early on illness days 1–3 to predict dengue disease outcome and the difference in CRP levels between dengue and other febrile illnesses (OFI).

**Method:**

We performed a nested case-control study using the clinical data and samples collected from the IDAMS-consortium multi-country study. This was a prospective multi-center observational study that enrolled almost 8000 participants presenting with a dengue-like illness to outpatient facilities in 8 countries across Asia and Latin America. Predefined severity definitions of severe and intermediate dengue were used as the primary outcomes. A total of 281 cases with severe/intermediate dengue were compared to 836 uncomplicated dengue patients as controls (ratio 1:3), and also 394 patients with OFI.

**Results:**

In patients with confirmed dengue, median (interquartile range) of CRP level within the first 3 days was 30.2 mg/L (12.4–61.2 mg/L) (uncomplicated dengue, 28.6 (10.5–58.9); severe or intermediate dengue, 34.0 (17.4–71.8)). Higher CRP levels in the first 3 days of illness were associated with a higher risk of severe or intermediate outcome (OR 1.17, 95% CI 1.07–1.29), especially in children. Higher CRP levels, exceeding 30 mg/L, also associated with hospitalization (OR 1.37, 95% CI 1.14–1.64) and longer fever clearance time (HR 0.84, 95% CI 0.76–0.93), especially in adults. CRP levels in patients with dengue were higher than patients with potential viral infection but lower than patients with potential bacterial infection, resulting in a quadratic association between dengue diagnosis and CRP, with levels of approximately 30 mg/L associated with the highest risk of having dengue. CRP had a positive correlation with total white cell count and neutrophils and negative correlation with lymphocytes, but did not correlate with liver transaminases, albumin, or platelet nadir.

**Conclusions:**

In summary, CRP measured in the first 3 days of illness could be a useful biomarker for early dengue risk prediction and may assist differentiating dengue from other febrile illnesses.

## Background

Dengue, caused by one of the four dengue virus serotypes (DENV1–4), is globally the most important arboviral infection, in terms of geographic spread and number of infections [1]. An estimated 390 million infections now occur annually in over 100 countries, of which 96 million manifest as symptomatic dengue cases [2]. The clinical phenotype can vary from a relatively mild self-limiting febrile illness, to severe and occasionally life-threatening symptoms of bleeding, organ impairment, and vascular leakage leading to shock [3]. These severe manifestations occur sufficiently late in the course of the disease around defervescence, which occurs usually on day 4–6 following illness onset, to allow a potential window of opportunity to identify patients who may progress.

In areas of dengue transmission, yearly seasonal epidemics occur and can very quickly overwhelm health facilities, with potentially thousands of patients being reviewed daily. As the vast majority of symptomatic infections will result in a benign disease course, the ability to identify patients at high risk of progression, who are likely to benefit from early intervention with supportive therapy, has become the focus of intense research efforts in recent years.

Several small studies have attempted to identify biomarkers for dengue that will be cost-effective in resource-limited settings [4]. Recent evidence suggests that markers of inflammation may be useful as biomarkers. Studies have shown higher levels of C-reactive protein (CRP) in severe dengue versus non-severe dengue, with a CRP cutoff level of 30.1 mg/L (AUC, 0.938; 100% sensitivity, 76.3% specificity) [5]. In adult patients in Indonesia on the third day of fever, CRP was higher in those who developed plasma leakage, 10.1 (IQR 4.3–36.5) vs. 6.3 (IQR 3.0–21.6) mg/L (*p* = 0.014) [6]. Other studies using highly sensitive (hs) CRP did not find a difference between the severity grades [7]. Higher levels of CRP have also been found in patients with dengue compared to other viral illnesses [8].

A lack of harmonization between these studies has made the results difficult to compare, with varying assay techniques, viral serotypes, age of the participants, immune status, and illness day at the time of sampling. To provide a definitive answer as to the utility of CRP measurement for diagnosis and risk prediction in dengue, these results require validation in a large sample set including early and dynamic sampling, and using a standardized assay.

We hypothesized that (1) dengue patients with higher CRP levels in the early febrile phase are at higher risk of developing severe disease and (2) dengue patients have higher CRP levels than patients with other viral febrile illnesses.

## Methods

### Study design

A nested case-control study was performed using the clinical data and blood samples already collected from patients recruited to the observational study entitled “Clinical evaluation of dengue and identification of risk factors for severe disease” (IDAMS study, NCT01550016), for which the protocol has been published [9]. Briefly, this prospective multi-center observational study enrolled 7428 participants aged 5 years or more, presenting with a febrile illness consistent with dengue to outpatient health facilities in 8 countries across Asia and Latin America. Patients at the participating sites were eligible for enrollment if they met the following criteria: fever or history of fever for less than 72 h and clinical symptoms consistent with possible dengue, and had no localizing features suggesting an alternative diagnosis, e.g., pneumonia. Participants were then followed daily until resolution of their acute illness, with a standard schedule of blood samples obtained during the illness course. Hospital admission and individual case management were determined according to clinical need, with all interventions documented in the case report forms. Subsequently, each participant was assigned an overall severity grading using all available information; the system used is described in detail in Additional file 1 and is in line with the recent recommendation to use standardized endpoints for severe and intermediate dengue to ensure reproducibility and comparability of research findings [10].

### Study population

**Fig. 1:**
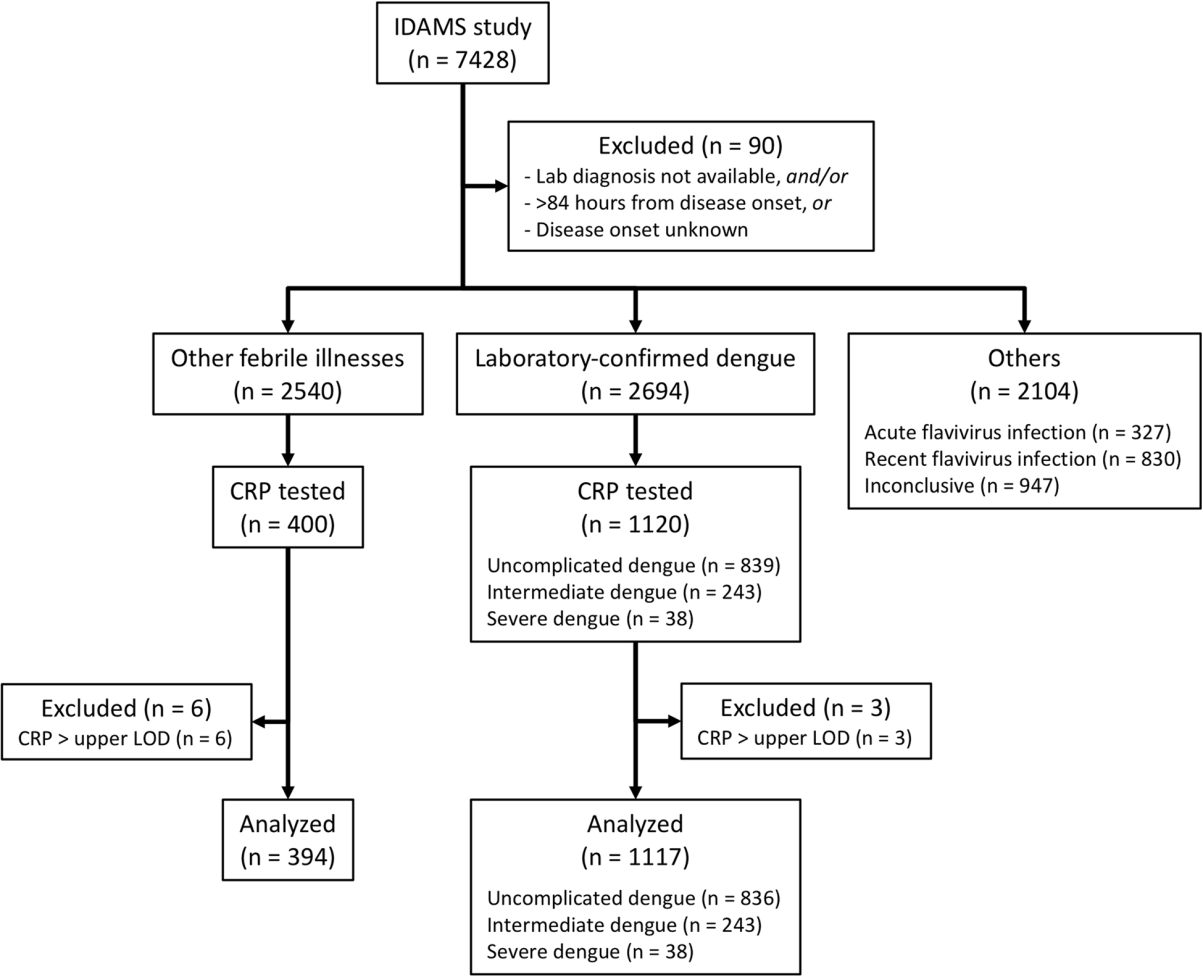
Study flowchart CRP, C-reactive protein; OFI, other febrile illnesses; LOD, limit of detection

Samples from participants enrolled at five study sites were selected, including Hospital for Tropical Diseases (HTD) (Ho Chi Minh City, Vietnam), National Hospital for Tropical Diseases (NHTD) (Hanoi, Vietnam), Angkor Hospital for Children (Siem Reap, Cambodia), University of Malaya Medical Centre (Kuala Lumpur, Malaysia), and Hospital Nacional de Niños Benjamin Bloom (El Salvador). Stored samples from patients who progressed to severe or intermediate dengue severity were selected as well as a comparison group of uncomplicated dengue and other febrile illnesses (OFI). This cohort included 38 severe and 243 intermediate severity cases (combined primary outcome of 281 cases). The 281 cases were compared to 839 uncomplicated dengue patients as controls (1:3), based on similar geographic and demographics and day of illness (DOI) making a total sample size of 1120 dengue cases. Confirmed dengue cases were compared to 400 patients with OFI, again from the same locations and with the same demographics and DOI. The study flowchart is shown in Fig. 1.

### Case definitions

Dengue diagnostic criteria have been described elsewhere; briefly, laboratory-confirmed dengue was defined by either a positive RT-PCR assay or a NS1 ELISA (Platelia NS1, Biorad) test [9]. Patients with no laboratory evidence of acute or recent dengue were assigned as OFI. For all dengue cases, immune status was classified based on capture IgG results. A probable primary infection was defined by negative IgG results on two consecutive specimens on the acute and early phases of dengue infection, with at least one specimen being obtained during the second week since the onset of symptom. A probable secondary infection was defined by any positive IgG result from acute or early samples [3, 9]. All other cases with the absence of suitable specimens at the appropriate time points were classified as inconclusive serology.

### Laboratory evaluation

A full blood count was performed daily, while biochemistry tests were only performed at enrollment and then subsequently if clinically indicated. A research sample of EDTA plasma was stored every other day. All research samples were processed at the different sites within 1 h of collection, centrifuged at 500*g*/min for 10 min, and then stored at − 20 °C. All the samples were transferred on dry ice to OUCRU laboratory. CRP was measured on these stored samples at two time points: enrollment sample (illness day 1–3) and follow-up (day 10–21 post-symptom onset) using the same commercial assay according to the manufacturer’s specifications (magnetic bead panel, cat. no. HCVD3MAG-67 K, Merck, Millipore, UK) on a Luminex 200 analyzer.

### Study endpoints

To compare CRP levels between dengue and OFI groups, the outcome was laboratory-confirmed dengue or not. To investigate the association between CRP levels and severity in dengue patients, the primary outcome was severe or intermediate dengue, which was defined as any of severe or intermediate plasma leakage, bleeding, neurology involvement, liver involvement, or other major organ failure; all definitions were in accordance with current standard definition for use in dengue interventional trials [10] (see Additional file 1). Severe dengue events were rare, and the combined endpoint of severe or intermediate dengue is relevant for clinical practice, since less severe manifestations that require medical intervention contribute disproportionately to disease burden. The decision to hospitalize is very subjective, so we included this as a secondary outcome. Other secondary outcomes included severe dengue or dengue with warning signs according to WHO 2009, and fever clearance time. Fever clearance time was defined as the number of days from symptom onset to the day of defervescence (for patients still febrile at hospital discharge, fever clearance time was censored at the day of discharge).

### Statistical analysis

Plasma CRP levels were transformed to base-2 logarithm (log 2) before analysis. Cox regression model was used to investigate fever clearance time while logistic regression model was used for other outcomes. The model comparing CRP levels between dengue and OFI groups was adjusted for age and day of illness (DOI) at enrollment. Other models for association between CRP levels and dengue severity were adjusted for age, DOI at enrollment, plasma viremia levels at enrollment, and immune status. Other factors, such as fluid infusion, bacteria coinfection, antibiotic usage, and comorbidity, had unclear potential relationship with either CRP level and clinical outcome, and therefore, they were not considered for the adjustment. As the sample size was unbalanced between day of illness and DOI 1 was unlikely to have a clinical event, we decided to adjust for DOI rather than to conduct a stratification analysis on this factor. The potentially non-linear effect of CRP levels on the outcomes was investigated using restricted cubic splines for log 2 of CRP levels with three knots at the 10th, 50th, and 90th percentiles [11]. The effect of CRP levels was expressed by odds ratio (OR) for the logistic regression models and hazard ratio (HR) for the Cox regression model. As the effect of CRP levels on the outcome could differ by age, we then performed a sensitivity analysis by subgroups of children (< 15 years of age) and adults (≥ 15 years of age), using similar statistical method as described above. Because the OFI group included both viral and bacterial infections, this group was further categorized into two subgroups using the level of neutrophil counts: cases with neutrophil counts of < 8 × 10^9^/mL (upper limit of normal) were classified as potential viral infection, and the others were classified as potential bacterial infection. CRP levels between dengue and these two subgroups were also explored using similar model. The association between CRP in dengue patients at enrollment and other clinical and biochemical markers (platelet nadir, maximum hematocrit (HCT) change, liver transaminases, creatinine kinase (CK), albumin, white blood cell (WBC) count, percentage of neutrophils and lymphocytes) were explored using scatter plots and Pearson’s correlation coefficients. All analyses were performed using the statistical software R version 3.4.4.

## Results

### Patient characteristics

Samples from 1120 patients with laboratory-confirmed dengue and 400 patients with OFI were tested with CRP. After excluding 9 samples with CRP values above the upper range of the assay, data from 1117 patients with laboratory-confirmed dengue and 394 patients with OFI were included in the final analysis (Fig. 1).

Due to the selection procedure, median age, gender, and DOI at enrollment were very similar between the OFI and dengue group (Table 1). The age range of all patients was 5 to 64 in the OFI group, and 5 to 73 in the dengue group. The number of adults and children enrolled by site can be seen in Additional file 2. Patients with severe dengue were younger (*p* < 0.001, Mann-Whitney *U* test) than those with other severity grades, and were more likely to be enrolled on day 3 (*p* = 0.003, chi-squared test). In the dengue patients, the percentage of secondary infections was highest in the severe dengue group 33/38 (86.8%) and lowest in the uncomplicated dengue group 542/836 (65.2%) (*p* < 0.001, chi-squared test). Fewer patients with OFI were hospitalized (76/394, 19.3%, versus 421/1120, 37.6%) (*p* < 0.001, chi-squared test). Within the OFI group, 131/394 (33.2%) of cases were clinically diagnosed as bacterial infection, while in the dengue group, there were 54/1120 (4.8%) of cases with clinical diagnosis of bacterial coinfection (see Additional file 3).

**Table 1:** Summary of clinical data

Characteristic	*n*	OFI (*N* = 394)	*n*	All dengue patients (*N* = 1117)	*n*	Uncomplicated dengue (*N* = 836)	*n*	Intermediate dengue (*N* = 243)	*n*	Severe dengue (*N* = 38)
Country	394		1117		836		243		38	
Cambodia		0 (0.0)		99 (8.9)		69 (8.3)		20 (8.2)		10 (26.3)
El Salvador		0 (0.0)		41 (3.7)		23 (2.8)		12 (4.9)		6 (15.8)
Malaysia		0 (0.0)		117 (10.5)		88 (10.5)		26 (10.7)		3 (7.9)
Vietnam		394 (100.0)		860 (77.0)		656 (78.5)		185 (76.1)		19 (50.0)
Age (year)	394	15 (8, 27)	1117	15 (10, 25)	836	14 (10, 25)	243	17 (11, 26)	38	10 (8, 14)
Gender male, *n* (%)	394	241 (61.2)	1117	637 (57.0)	836	467 (55.9)	243	147 (60.5)	38	23 (60.5)
DOI at enrollment, *n* (%)	394		1117		836		243		38	
1		99 (25.1)		225 (20.1)		176 (21.1)		42 (17.3)		7 (18.4)
2		162 (41.1)		555 (49.7)		425 (50.8)		117 (48.1)		13 (34.2)
3		133 (33.8)		337 (30.2)		235 (28.1)		84 (34.6)		18 (47.4)
Serotype, *n* (%)	–		1049		782		231		36	
DENV-1		–		435 (41.5)		314 (40.2)		101 (43.7)		20 (55.6)
DENV-2		–		186 (17.7)		139 (17.8)		42 (18.2)		5 (13.9)
DENV-3		–		105 (10.0)		76 (9.7)		26 (11.3)		3 (8.3)
DENV-4		–		323 (30.8)		253 (32.4)		62 (26.8)		8 (22.2)
Serology, *n* (%)	–		1117		836		243		38	
Probable primary		–		215 (19.2)		174 (20.8)		39 (16.0)		2 (5.3)
Probable secondary		–		763 (68.3)		545 (65.2)		185 (76.1)		33 (86.8)
Inconclusive		–		139 (12.4)		117 (14.0)		19 (7.8)		3 (7.9)
Used antibiotics^1^, *n* (%)	392	140 (35.7)	1115	74 (6.6)	834	46 (5.5)	243	20 (8.2)	38	8 (21.1)
DOI of starting antibiotic	139	4.0 (3.0, 5.0)	74	4.0 (3.0, 5.8)	46	4.0 (3.0, 5.0)	20	4.0 (3.0, 6.0)	8	6.5 (4.0, 8.0)
Length of fever (day)^2^	344	5.0 (5.0, 6.0)	914	6.0 (5.0, 7.0)	708	6.0 (5.0, 7.0)	184	7.0 (6.0, 7.0)	22	7.5 (6.0, 8.0)
Used any blood product^3^, *n* (%)	392	0 (0.0)	1117	3 (0.3)	836	0 (0.0)	243	2 (0.8)	38	1 (2.6)
Hospitalization, *n* (%)	394	76 (19.3)	1117	421 (37.7)	836	260 (31.1)	243	123 (50.6)	38	38 (100.0)
DOI when hospitalization	76	4.0 (3.0, 5.0)	421	5.0 (4.0, 5.0)	260	4.0 (4.0, 5.0)	123	5.0 (4.0, 5.0)	38	4.5 (4.0, 5.0)
Length of hospital stay (day)	76	4.0 (4.0, 6.0)	420	4.0 (3.0, 5.0)	260	3.0 (2.0, 4.0)	123	4.0 (3.0, 5.0)	37	5.0 (4.0, 6.0)
Clinical diagnosis of bacterial infection, *n* (%)	394	131 (33.2)	1117	54 (4.8)	836	38 (4.5)	243	12 (4.9)	38	4 (10.5)
Used antibiotics^4^, *n* (%)	131	113 (86.3)	54	41 (75.9)	38	29 (76.3)	12	9 (75.0)	4	3 (75.0)

Summary statistic is median (interquartile range) for numeric variables and absolute count (percentage) for categorical variables

*DOI* day of illness, *OFI* other febrile illnesses

^1^There were 4 missing cases about the information of using antibiotic or not (2 in the OFI group and 2 in the uncomplicated dengue group)

^2^There were 253 cases with fever at the end of study follow-up; therefore, the time of defervescence was unknown

^3^There were 2 cases received platelet transfusion in the intermediate dengue group, and 1 case received whole blood transfusion in the severe dengue group

^4^Summary data of using antibiotics in each probable bacterial group

### Laboratory characteristics

**Fig. 2:**
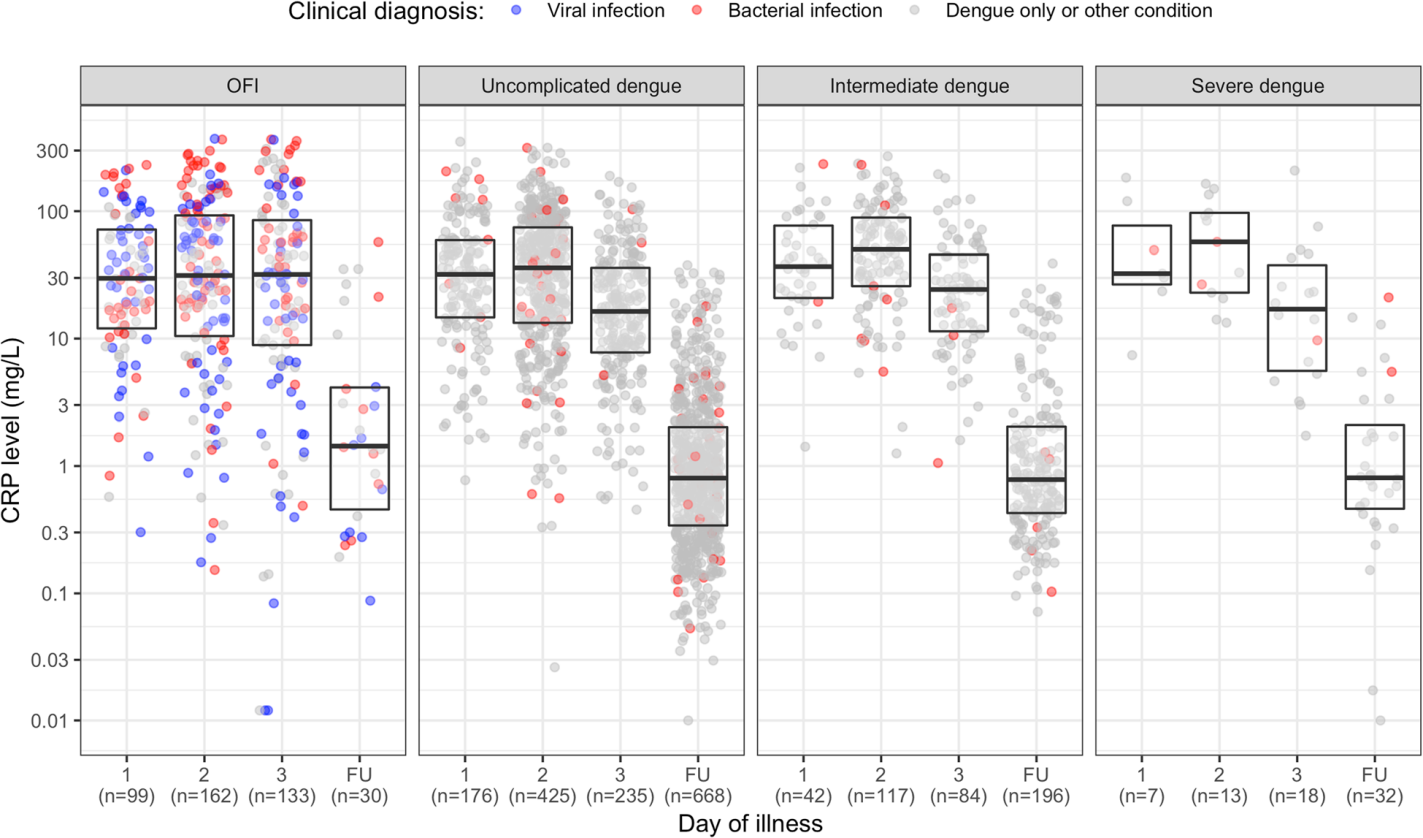
Summary of CRP levels by day of illness at enrollment and follow-up The upper and lower edges of each box represent the interquartile range (25th–75th percentile) while the middle line is corresponding to the median. The points are the actual CRP values and colored by clinical diagnosis of bacterial infection (in red), viral infection (in blue), and dengue only infection (for patients with dengue infection) or other condition (for patients in the OFI group) (in gray). Among patients in the OFI group, 131 were clinically bacterial infection, 139 were clinically viral infection, and 124 were other conditions. The day of illness at enrollment is 1, 2, or 3, and FU is the follow-up period. The *y*-axis is transformed using base-2 logarithm. CRP, C-reactive protein; FU, follow-up; OFI, other febrile illnesses

In patients with dengue infection, median CRP level at enrollment was 30.2 mg/L, similar to the OFI group (median CRP of 31.3) (Table 2). In comparing DOI at enrollment, CRP level was highest in patients enrolled on DOI 2 and lower in patients enrolled on DOI 3 or at follow-up. This trend is similar across dengue severity (Fig. 2). There were 30 patients in the OFI group and 896 patients in the dengue group who had CRP measurements at follow-up (Table 2, Fig. 2). The follow-up days ranged from 10 to 31 days; however, 94% follow-up samples were within 10–20 days since onset. For the OFI group, while the same trend was seen in patients clinically diagnosed with bacterial infection, CRP level among patients with viral diagnosis was lower for those enrolled at a later time point. Considering the other laboratory parameters, dengue patients had higher levels of liver transaminases and CK, but lower WBC, and comparable levels of albumin (Table 2). Dengue patients had higher maximum HCT change, but lower PLT nadir. Among patients with dengue, plasma viremia levels were highest in the severe group.

**Table 2:** Summary of laboratory data

Characteristic	*n*	OFI (*N* = 394)	*n*	All dengue patients (*N* = 1117)	*n*	Uncomplicated dengue (*N* = 836)	*n*	Intermediate dengue (*N* = 243)	*n*	Severe dengue (*N* = 38)
At enrollment										
CRP (mg/L)	394	31.3 (10.2, 88.8)	1117	30.2 (12.4, 61.5)	836	28.6 (10.5, 58.9)	243	35.3 (17.9, 71.8)	38	26.2 (14.1, 71.1)
Viremia level (× 10^6^ copies/mL)	–	–	1050	35.5 (3.1, 269.6)	783	25.2 (2.0, 185.4)	231	92.9 (7.5, 690.5)	36	128.5 (33.1, 331.2)
AST (UI/L)	394	30.0 (23.0, 40.0)	1109	43.0 (30.0, 67.0)	831	42.0 (30.0, 62.0)	240	52.0 (33.0, 92.2)	38	60.0 (43.0, 84.8)
ALT (UI/L)	394	18.0 (13.0, 30.0)	1113	28.0 (18.0, 49.0)	834	27.0 (17.0, 46.0)	241	32.0 (21.0, 67.0)	38	29.0 (18.2, 41.2)
Albumin (g/L)	394	45.5 (43.5, 47.5)	1105	45.0 (42.5, 47.1)	828	45.0 (42.7, 47.3)	239	44.3 (41.9, 46.9)	38	44.9 (42.6, 47.3)
Creatine kinase (UI/L)	394	92.5 (70.2, 129.0)	1012	105.0 (77.8, 156.2)	763	104.0 (76.0, 149.0)	221	115.0 (83.0, 171.0)	28	138.0 (91.0, 270.0)
WBC (× 10^9^/mL)	394	7.7 (5.5, 10.8)	1113	4.3 (3.2, 6.0)	834	4.3 (3.2, 6.0)	241	4.4 (3.1, 6.0)	38	4.0 (2.8, 4.9)
Neutrophils (%)	394	71.0 (61.5, 79.0)	1108	67.5 (58.0, 75.2)	830	66.3 (57.0, 74.8)	240	70.0 (61.4, 78.0)	38	72.8 (60.3, 77.9)
Lymphocytes (%)	394	16.5 (11.5, 24.1)	1109	18.6 (11.8, 26.9)	831	18.9 (12.4, 27.4)	240	16.9 (10.9, 24.4)	38	19.8 (11.8, 31.3)
Other time points										
Max HCT change* (%)	360	2.4 (− 1.5, 5.8)	1112	7.1 (2.4, 13.7)	832	5.4 (1.7, 9.9)	243	20.5 (8.6, 24.5)	37	16.7 (6.1, 28.2)
Platelet nadir (× 10^9^/mL)	383	178 (143, 225)	1113	72.0 (40.0, 119.0)	834	89 (52, 127)	242	40.5 (22.0, 71.0)	37	18 (13, 30)
CRP at follow-up (mg/L)	30	1.4 (0.5, 4.1)	896	0.8 (0.4, 2.0)	668	0.8 (0.3, 2.0)	196	0.8 (0.4, 2.0)	32	0.8 (0.5, 2.2)

Summary statistics are median (interquartile range)

*ALT* alanine aminotransferase, *AST* aspartate aminotransferase, *CRP* C-reactive protein, *DOI* day of illness, *HCT* hematocrit, *OFI* other febrile illnesses, *WBC* white blood cell

*Maximum HCT change was calculated by maximum HCT level at the acute phase (DOI 4 to 7) minus baseline HCT, then divided by baseline HCT. Baseline HCT was the minimum HCT level within DOI 1 to 3

### Association of CRP levels and dengue diagnosis

**Fig. 3:**
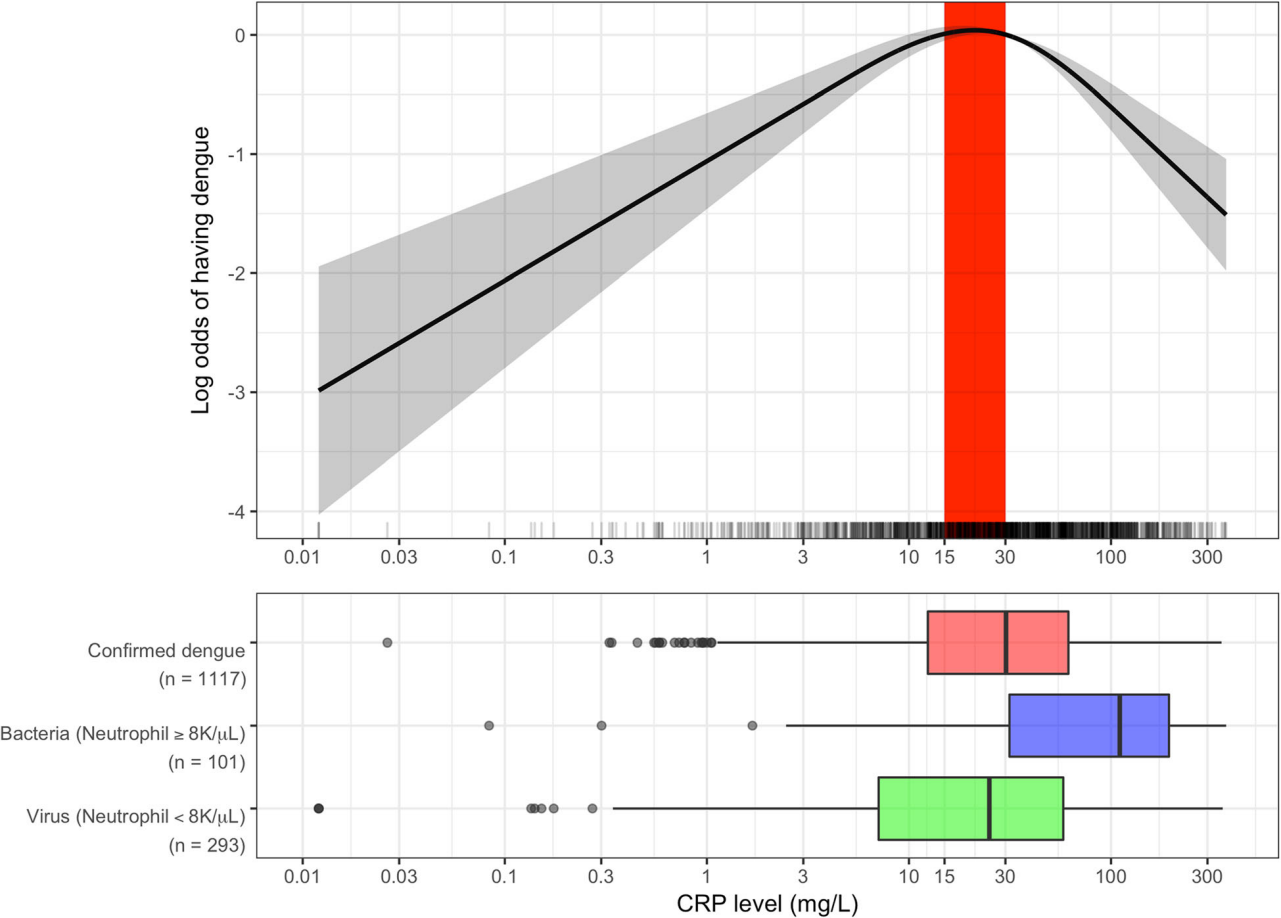
Association between plasma CRP level and patients diagnosed with dengue or OFI The log odds of having dengue (the black line) and its 95% confidence interval (the gray region) by CRP level were estimated from multivariable logistic regression models with non-linear effect of log 2 of CRP levels, which modeled using restricted cubic splines with 3 knots, and adjustment for age and DOI at enrollment. The red region highlights the range of CRP of 15–30 mg/L, which corresponds to the highest probability of having dengue. The rug plots on the *x*-axis represent the distribution of CRP value of individual cases. Horizontal plots described distribution of CRP levels among dengue group (in red) and OFI group (the OFI group was further separated into potential bacterial infection [in blue] and potential viral infection [in green] based on clinical diagnosis by treating doctor [clinical diagnosis] or number of neutrophil). There are significant differences between CRP levels in the dengue group with the potential bacterial infection group (*p* < 0.001) and with the potential viral infection group (*p* = 0.003) (Mann-Whitney *U* test). The *x*-axis was transformed using base-2 logarithm. CRP, C-reactive protein; DOI, day of illness; OFI, other febrile illnesses

The OFI group was separated into potential viral or bacterial infections, using the clinical diagnosis and a neutrophil cutoff of 8 × 10^9^/mL. The boxplots (lower part of Fig. 3) suggest that CRP levels in dengue patients are slightly higher than in patients with probable viral infection (*p* = 0.003, Mann-Whitney *U* test) but lower than in patients with probable bacterial infection (*p* < 0.001, Mann-Whitney *U* test). These differences resulted in a non-linear relationship between dengue diagnosis and CRP levels, as depicted in the upper plot in Fig. 3. CRP levels of less than 30 mg/L (approximately the median CRP levels in dengue patients) showed that higher CRP levels associated with higher odds of dengue diagnosis (OR for each two times increase in CRP level was 1.28, 95% CI 1.18–1.40). However, in patients with CRP levels ≥ 30 mg/L, higher CRP associated with lower odds of dengue diagnosis (OR for each two times increase in CRP level was 0.64, 95% CI 0.55–0.73). The subgroup analysis of age < 15 and age ≥ 15 years showed similar results (see Additional file 4). A sensitivity analysis showed that including the 9 cases with CRP values above the upper range of the assay did not affect the results (ORs [95% CIs] in patients with CRP levels < 30 and ≥ 30 mg/L were 1.28 [1.18–1.40] and 0.63 [0.55–0.72], respectively).

### Association of CRP level and clinical outcomes among dengue patients

**Fig. 4:**
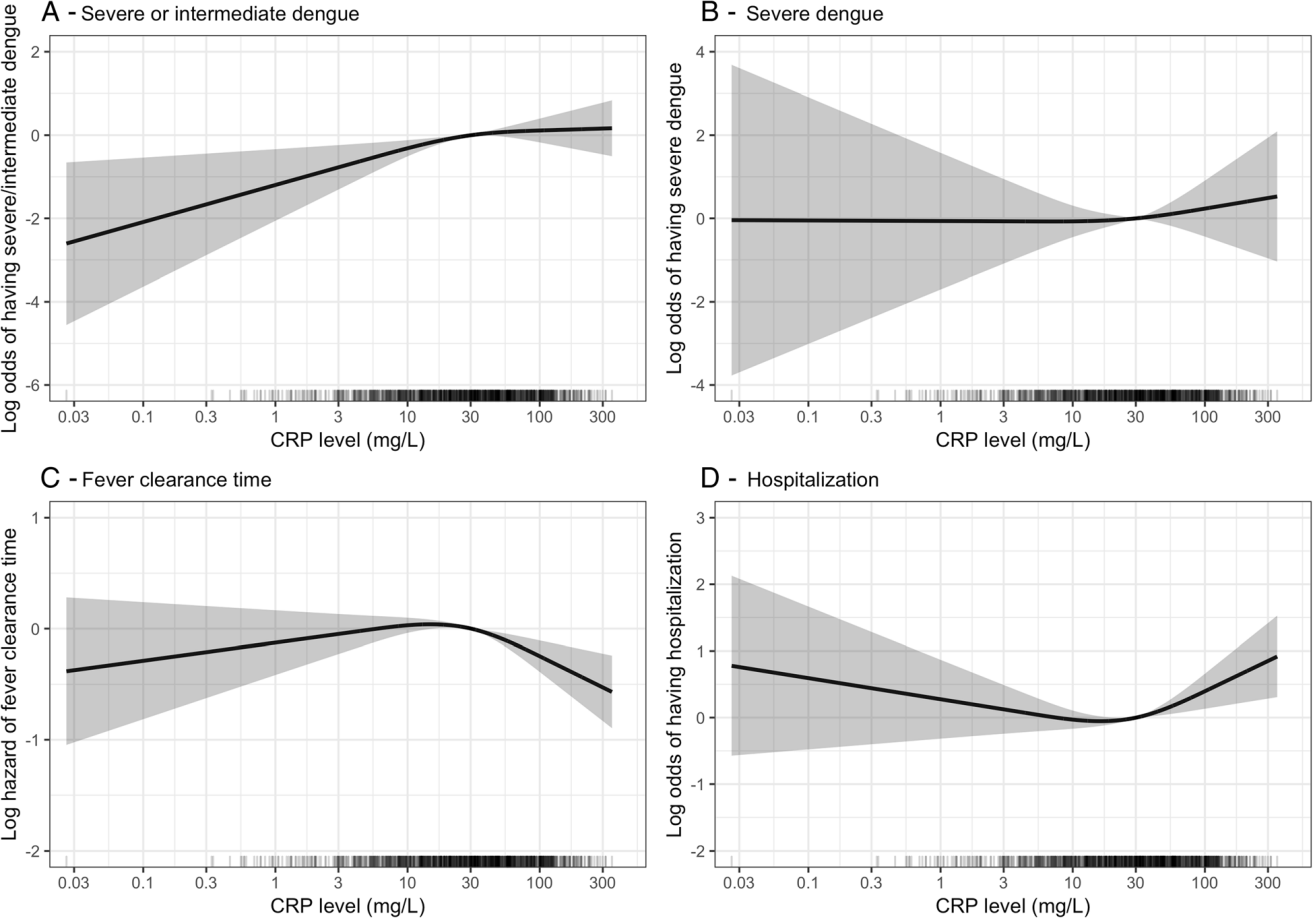
**a**–**d** Association between CRP level and clinical outcomes among dengue patients The log odds (or log hazard for fever clearance time) of the outcomes (the black line) and its 95% confidence interval (the gray region) are estimated from multivariable logistic regression models (or multivariable Cox model for fever clearance time) allowing for non-linear effect of log 2 of CRP levels using restricted cubic splines and adjusted for age, DOI at enrollment, viremia levels at enrollment, and immune status. The rug plot on the *x*-axis represents the distribution of individual cases. The *x*-axis is transformed using base-2 logarithm. CRP, C-reactive protein

The median (IQRs) DOIs of developing the outcomes of intermediate and severe dengue were 5 (4; 6) and 5 (5; 6), and median (IQR) times from the CRP test to these outcomes were 3 (2; 4) and 3 (2; 4) days, respectively. CRP levels at enrollment in patients with severe or intermediate dengue were higher than in patients with uncomplicated dengue, with median levels (interquartile range) of 34.0 (17.4–71.8 mg/L), and 28.6 mg/L (10.5–58.9 mg/L), respectively. Higher CRP levels in the first 3 days of illness were associated with increased odds of severe or intermediate dengue, after correcting for age, DOI at enrollment, plasma viremia levels, and immune status (Table 3). For each twofold increase in CRP level, the OR (95% CI) of having severe or intermediate dengue was 1.17 (1.07–1.29). Patients with higher CRP level also had higher odds of severe dengue with OR of 1.05 (95% CI 0.85–1.32), but this did not reach statistical significance. Higher CRP levels in patients with CRP ≥ 30 mg/L were strongly associated with longer fever clearance time (HR 0.84, 95% CI 0.76–0.93) and hospitalization (OR 1.37, 95% CI 1.14–1.64) (Table 3, Fig. 4). When categorizing CRP levels into 3 groups (< 15, 15–30, and ≥ 30 mg/L), the 2 latter groups had significantly higher risk of severe or intermediate dengue compared to the lowest CRP level group (ORs [95% CIs] were 1.66 [1.07–2.56] and 1.59 [1.10–2.32], respectively), after correcting for age, DOI at enrollment, plasma viremia, and immune status.

**Table 3:** Association between CRP level and clinical outcomes among dengue patients

Outcome	Crude OR/HR	95% CI	*p* value	Adjusted OR/HR	95% CI	*p* value
Severe or intermediate dengue^1^	1.17	1.08–1.27	< 0.001	1.18	1.07–1.30	0.001
Severe dengue^1^	1.04	0.87–1.25	0.710	1.05	0.85–1.32	0.638
Fever clearance time*^2^						
CRP < 30 mg/L	1.00	0.94–1.05	0.866	1.03	0.97–1.09	0.387
CRP ≥ 30 mg/L	0.89	0.81–0.97	0.010	0.84	0.76–0.92	< 0.001
Hospitalization^2^						
CRP < 30 mg/L	0.93	0.83–1.03	0.159	0.92	0.81–1.04	0.175
CRP ≥ 30 mg/L	1.28	1.08–1.51	0.004	1.36	1.13–1.63	0.001

The estimates (OR and HR) and 95% CI were reported for each one log 2 increase of CRP level, i.e., for each 2 times increase of CRP level

*CI* confidence interval, *CRP* C-reactive protein, *DOI* day of illness, *HR* hazard ratio, *OR* odds ratio

*We use hazard ratio (HR) to report the results of Cox model for fever clearance time outcome. All other outcomes are reported by odds ratio (OR) estimated from logistic regression model. All adjusted estimates are derived from multivariable models adjusted for age, DOI at enrollment, plasma viremia level, and immune status

^1^The models for severe or intermediate dengue and severe dengue were performed with linear effect of log 2 of CRP (the non-linear effect of log 2 of CRP was not statistically significant: *p* = 0.209 for severe or intermediate dengue outcome, and *p* = 0.679 for severe dengue outcome)

^2^The models for fever clearance time and hospitalization were performed with two separated linear effect of log 2 of CRP (for CRP < 30 and CRP ≥ 30 mg/L). The non-linear effect of log 2 of CRP was statistically significant: *p* = 0.004 for fever clearance time outcome, and *p* = 0.011 for hospitalization outcome. These models had a quadratic effect with the peak CRP level of approximately 30 mg/L

The subgroup analysis by age, regarding severe or intermediate dengue, showed similar results in children (OR 1.18, 95% CI 1.04–1.35); however, the magnitude of effect was less in adults (OR 1.10, 95% CI 0.96–1.27) (see Additional files 5 and 6). The strong association between higher CRP level and longer fever clearance time and hospitalization when CRP level ≥ 30 mg/L was still apparent in adults (HR of fever clearance was 0.78, 95% CI 0.68–0.89; OR of hospitalization was 1.59, 95% CI 1.25–2.04) but not in children (see Additional files 5, 7, and 8). A sensitivity analysis including 9 cases with CRP values above the upper range of the assay also showed similar results (see Additional file 9).

Overall, antibiotics were used in 140/392 (35.7%) patients in the OFI group but only in 74/1118 (6.6%) patients in the dengue group. Among 428 dengue patients with CRP level at enrollment of > 40 mg/L and without clinically suspected bacterial infection, 417 (97%) patients did not receive antibiotics (12 from severe dengue group, 107 from intermediate dengue group, and 298 from uncomplicated dengue group).

### Association between CRP level and other laboratory tests among dengue patients

**Fig. 5:**
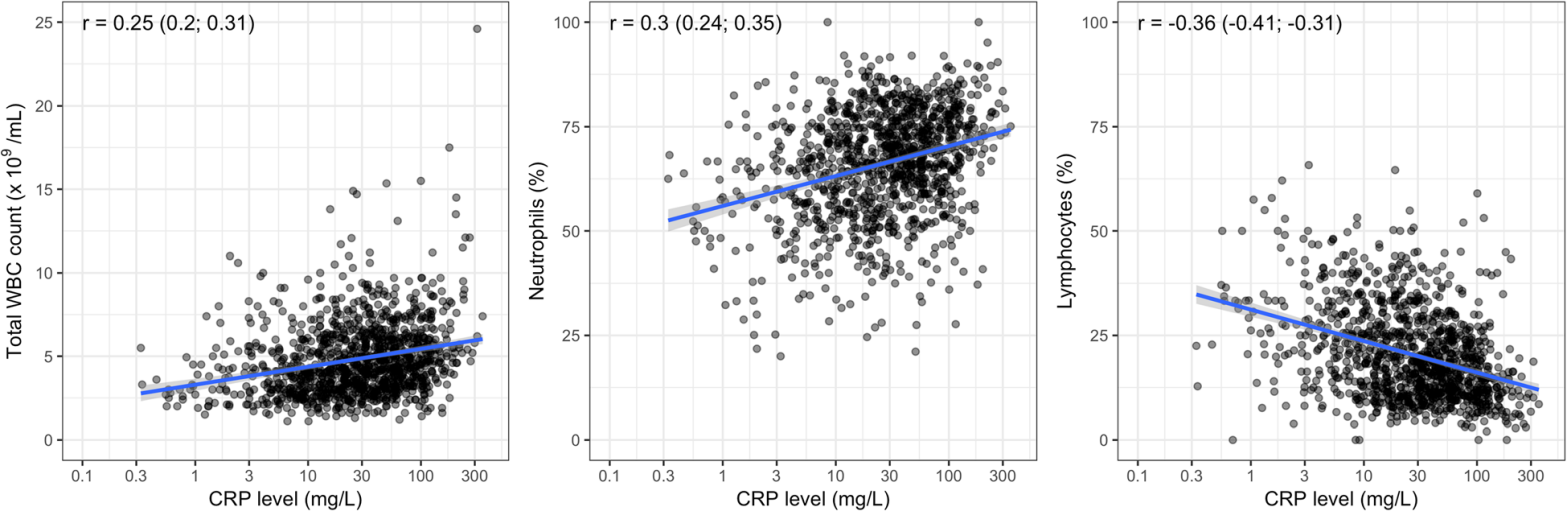
Association between CRP and total white blood cell count (*n* = 1115), the percentages of neutrophils (*n* = 1110) and lymphocytes (*n* = 1111) Each black point represents for each patient. The blue line is the linear regression line, and the gray region is the 95% confidence interval. Pearson’s correlation coefficient and its 95% confidence interval are shown in the top left corner of each plot. The *x*-axis is transformed using base-2 logarithm. CRP, C-reactive protein; WBC, white blood cell

Among dengue patients, we found positive associations between CRP levels and total WBC count and the neutrophil percentage (Pearson’s correlation coefficients were 0.25 and 0.3, respectively), and a negative association between CRP and the lymphocyte percentage (Pearson’s correlation coefficient was − 0.36) (Fig. 5). There were no significant associations between CRP level and other laboratory tests including liver transaminases, CK, albumin, maximum HCT change, and PLT nadir (see Additional files 10 and 11).

## Discussion

This large case-control study has shown that increased CRP levels in the first 3 days of illness are associated with worse dengue clinical outcomes, especially in children. These findings provide the most robust evidence of this relationship to date, with a consistent approach across a large and diverse multi-national cohort ensuring maximal generalisability. In identifying an association between higher CRP and a modest increase in the likelihood of progression to intermediate/severe disease, we have demonstrated the potential utility of CRP measured early in the course of infection. This observation could assist patient management at the point of initial presentation, when accurate triage of those most in need of supportive care is imperative. Existing WHO criteria for dengue with warning signs rely heavily on clinical examination findings, with the inclusion of changes in hematocrit and platelet count representing the only laboratory markers of anticipated severity [3]. However, while the importance of these parameters has been emphasized by recent studies [12–14], our findings suggest that the approach to risk stratification could be further refined with the inclusion of baseline CRP.

The prognostic potential of markers of generalized inflammation has previously been suggested, in view of the known immune-enhancing effect inherent to dengue’s pathogenicity [4, 15]. However, recent investigation into the association between CRP and severity of disease has yielded conflicting results [5, 6, 16–18]. Our findings are consistent with those of a smaller study of 191 adults, which reported higher levels of CRP during the febrile phase of illness in severe versus non-severe dengue (defined using 2009 WHO classification) (AUC 0.938 at a CRP threshold of 30.1 mg/L; 100% sensitivity, 76.3% specificity) [5]. Other studies have shown a lower range of CRP in dengue infection, but this variability is likely reflective of heterogeneity in laboratory methods used, timing of serum sampling, and classification of clinical outcome. Importantly, each study reporting an absence of association between CRP and severity of disease focused on measures of CRP taken later than 3 days into the course of illness, which may account for their disagreement with our results [16, 17].

CRP is an acute-phase protein that is rapidly synthesized by hepatocytes in response to inflammatory stimuli. It binds to a number of intrinsic and extrinsic ligands, including many constituents of eukaryotic and prokaryotic pathogens, enabling activation of the classical complement pathway and playing a possible role in alternative-pathway regulation [19]. While the mechanism of association between elevated CRP and dengue remains unclear, immune enhancement is a known feature of dengue pathogenesis, and it may be that severity of disease manifestation is determined by overall magnitude of the immune response. The observed association between higher CRP and increased probability of increased disease severity should serve to inform future clinical trial design, both by enriching study populations and by encouraging exploration of potential anti-inflammatory host-directed therapeutics.

Aside from CRP, several other components of the immune response have been identified as potential prognostic biomarkers [4]. Nascimento et al. found associations between markers of dysregulation of the alternative complement pathway and the development of dengue haemorrhagic fever [16], and Juffrie et al. reported levels of the proinflammatory molecules, IL-6 and sPLA2, to be predictive of dengue-associated hypotensive shock [18]. Additional proposed prognostic indicators include a range of proinflammatory cytokines, markers of endothelial activation and microvascular disruption, and acute-phase proteins, but the evidence base to support these associations is limited to small studies with multiple possible confounding sources [4, 17, 20]. The major distinguishing feature that separates CRP from other potential immune biomarkers, however, is its well-established use as a clinical test, with cheap point-of-care (POC) kits readily available across the majority of healthcare settings. It is this ease of access, which makes it a highly attractive option for implementation as a prognostic tool in the resource-limited areas of greatest dengue disease burden.

In addition to addressing the important question of whether CRP can be prognostically useful as a dengue biomarker, our findings add to the existing evidence base for its use in differentiating dengue from non-dengue causes of fever [8, 21–30]. We found the median CRP level of approximately 30 mg/L among dengue cases within the first 3 days of illness. Other studies found similar levels with observed mean CRP levels of 19.0 mg/L on the first day of illness in dengue cases [23]. A number of previous studies have demonstrated the role of CRP in differentiating dengue from specifically bacterial [21–26] and malarial infections [25–28], although the most effective CRP thresholds for predicting underlying etiology remain difficult to define. Furthermore, the use of a CRP threshold of 20 mg/L has been demonstrated in simulation models to be successful and cost-effective in classifying patients into those that would and would not benefit from antibiotics in 80% of cases, regardless of baseline endemicity levels of different pathogens [24]. In practice, the use of POC CRP assay has been shown to reduce antibiotic prescribing when implementing thresholds of significance of both 20 mg/L and 40 mg/L [31]. However, our results highlight the risk of inadvertently increasing inappropriate antibiotic prescribing if dengue is not first considered in patients with CRP > 20 mg/L during seasons of high incidence. Nevertheless, growing confidence in the diagnostic value of CRP makes POC CRP an increasingly relevant component of antimicrobial stewardship strategy in the present era of ever-growing antimicrobial resistance.

It is important to acknowledge some limitations of our nested case-control study design, with observations relating to the diagnostic value of CRP in dengue infection being particularly susceptible to confounding by selection bias. For example, uncomplicated cases were chosen to match demographic characteristics of the severe/intermediate group, so the CRP results from these uncomplicated cases cannot be generalized to all uncomplicated dengue cases in the cohort. This effect would be minimized by further exploration in future large prospective cohort studies, including more patients from Latin America. The absence of available data on alternative causes of febrile illness in the study population also makes it difficult to draw firm conclusions about the differentiating power of CRP.

## Conclusions

Despite increasing global efforts to reduce the physical and socioeconomic impact of dengue, reliable biomarkers for predicting disease severity remain scarce. This study provides important insight into the association between higher CRP and risk of disease progression, with results that are generalisable across the range of populations most commonly affected. These findings can be used to influence further research design and health policy on how best to risk-stratify patients at the point of initial assessment.

## Supplementary information

**Additional file 1.** Clinical endpoint definition.

**Additional file 2.** Number of adults/children by sites.

**Additional file 3.** Summary of clinically diagnosis of bacterial infection.

**Additional file 4: Figure S1.** Association between CRP level and patients diagnosed with dengue or OFI.

**Additional file 5.** Association between CRP level and clinical outcomes in subgroups of age <15 years and age ≥15 years.

**Additional file 6: Figure S2.** Association between CRP level and severe or intermediate dengue among dengue patients.

**Additional file 7: Figure S3.** Association between CRP level and fever clearance time among dengue patients.

**Additional file 8: Figure S4.** Association between CRP level and hospitalization among dengue patients.

**Additional file 9.** Association between CRP level and clinical outcomes: sensitivity analysis including 9 cases with CRP values above the upper range of the assay.

**Additional file 10: Figure S5.** Association between CRP and other laboratory tests result.

**Additional file 11.** Correlation between CRP and other biomarkers.

## Data Availability

Dataset available from the OUCRU repository—on request to the corresponding author (S Yacoub).

## Abbreviations

ALT: Alanine aminotransferase
AST: Aspartate aminotransferase
CK: Creatinine Kinase
CRP: C-reactive protein
DENV: Dengue Virus
DOI: Day of illness
FU: Follow-up
HCT: Haematocrit
HR: Hazard Ratio
HTD: Hospital for Tropical Diseases
IQR: Interquartile range
LOD: Limit of detection
OFI: Other Febrile illness
OR: Odds Ratio
PLT: Platelet
POC: Point of care
RT-PCR: Real-time Polymerase chain reaction
WBC: White blood cell
