# Supplementary material for: C-reactive protein as a potential biomarker for disease progression in dengue: a multi-country observational study
Source: BMC Med. 2020 Feb 17;18:35. doi: 10.1186/s12916-020-1496-1 (PMC7025413; doi:10.1186/s12916-020-1496-1)
Supplement: Supplementary file 1 — Additional file 1. Clinical endpoint definition. [file 12916_2020_1496_MOESM1_ESM.docx]

**Additional file 1: Clinical endpoint definition**

| **Endpoint** | **Definition** |
| --- | --- |
| Severe plasma leakage | Dengue shock syndrome or respiratory distress due to plasma leakage |
| Intermediate plasma leakage | Did not fulfill criteria for severe plasma leakage and had at least one of the following criteria: (1) maximum HCT change was 20% or more, and (2) having evidence of fluid accumulation |
| Severe bleeding | Any bleeding into a critical organ or required any blood transfusion of packed red cells or whole blood without pre-anemia, or bleeding with complication |
| Intermediate bleeding | Did not fulfill criteria for severe bleeding and had at least one of the following criteria: (1) severe bleeding by clinical judgement, (2) bleeding required any blood transfusion other than packed red cells or whole blood, (3) bleeding required other intervention (e.g. nasal packing, cross-match, etc.), and (4) receiving packed red cells or whole blood with pre-existing anaemia and with a consistent haemoglobin value |
| Severe neurologic involvement | Abnormal neurologic examination and neurologic involvement that resulted in death or ongoing sequelae that impaired daily function, or required intubation, shunting or intensive care |
| Intermediate neurology involvement | Single convulsion without hospitalization or other complication |
| Severe hepatic involvement | Jaundice or coagulopathy or encephalopathy |
| Intermediate hepatic involvement | Any ALT or AST result of 400 IU/L or more |
| Severe other major organ failure | CK or other enzymes (e.g. troponin) abnormalities and functional abnormalities (e.g. reduced cardiac ejection fraction less than 50% or new ECG abnormalities) or required specific intervention (e.g. inotropic support) |
| Intermediate other major organ failure | Troponin abnormalities alone or CK abnormalities without cardiac ejection fraction less than 50% |
