# Supplementary material for: C-reactive protein as a potential biomarker for disease progression in dengue: a multi-country observational study
Source: BMC Med. 2020 Feb 17;18:35. doi: 10.1186/s12916-020-1496-1 (PMC7025413; doi:10.1186/s12916-020-1496-1)
Supplement: Supplementary file 2 — Additional file 2. Number of adults/children by sites. [file 12916_2020_1496_MOESM2_ESM.docx]

**Additional file 2: Number of adults/children by sites**

|  | All patients (N=1511) | Cambodia (N=99) | El Salvador (N=41) | Malaysia (N=117) | Vietnam (N=1254) |
| --- | --- | --- | --- | --- | --- |
| Children | 742 (49.1%) | 95 (96.0%) | 41 (100.0%) | 7 (6.0%) | 599 (47.8%) |
| Adults | 769 (50.9%) | 4 (4.0%) | 0 (0.0%) | 110 (94.0%) | 655 (52.2%) |
