# Supplementary material for: C-reactive protein as a potential biomarker for disease progression in dengue: a multi-country observational study
Source: BMC Med. 2020 Feb 17;18:35. doi: 10.1186/s12916-020-1496-1 (PMC7025413; doi:10.1186/s12916-020-1496-1)
Supplement: Supplementary file 3 — Additional file 3. Summary of clinically diagnosis of bacterial infection. [file 12916_2020_1496_MOESM3_ESM.docx]

**Additional file 3: Summary of clinical diagnosis of bacterial infection**

| Clinical bacterial diagnosis | OFI  (N=131) | All dengue patients  (N=54) | Uncomplicated dengue  (N=38) | Intermediate dengue  (N=12) | Severe dengue  (N=4) |
| --- | --- | --- | --- | --- | --- |
| Abscess | 2 (1.5%) | 0 (0.0%) | 0 (0.0%) | 0 (0.0%) | 0 (0.0%) |
| Acute bronchitis | 8 (6.1%) | 2 (3.7%) | 2 (5.3%) | 0 (0.0%) | 0 (0.0%) |
| Adenitis | 0 (0.0%) | 1 (1.9%) | 1 (2.6%) | 0 (0.0%) | 0 (0.0%) |
| Appendicitis | 1 (0.8%) | 0 (0.0%) | 0 (0.0%) | 0 (0.0%) | 0 (0.0%) |
| Gastrointestinal infection | 2 (1.5%) | 3 (5.6%) | 2 (5.3%) | 1 (8.3%) | 0 (0.0%) |
| Gingivitis | 1 (0.8%) | 1 (1.9%) | 1 (2.6%) | 0 (0.0%) | 0 (0.0%) |
| Impetigo | 0 (0.0%) | 1 (1.9%) | 1 (2.6%) | 0 (0.0%) | 0 (0.0%) |
| Infected wound | 0 (0.0%) | 1 (1.9%) | 1 (2.6%) | 0 (0.0%) | 0 (0.0%) |
| Infectious diarrhoea | 1 (0.8%) | 0 (0.0%) | 0 (0.0%) | 0 (0.0%) | 0 (0.0%) |
| Meningitis | 0 (0.0%) | 1 (1.9%) | 0 (0.0%) | 0 (0.0%) | 1 (25.0%) |
| Pharyngitis | 65 (49.6%) | 17 (31.5%) | 15 (39.5%) | 2 (16.7%) | 0 (0.0%) |
| Pneumonia | 8 (6.1%) | 1 (1.9%) | 0 (0.0%) | 0 (0.0%) | 1 (25.0%) |
| Pulmonary tuberculosis | 1 (0.8%) | 1 (1.9%) | 0 (0.0%) | 1 (8.3%) | 0 (0.0%) |
| Respiratory infection | 21 (16.0%) | 9 (16.7%) | 8 (21.1%) | 1 (8.3%) | 0 (0.0%) |
| Sepsis | 4 (3.1%) | 3 (5.6%) | 1 (2.6%) | 1 (8.3%) | 1 (25.0%) |
| Sinusitis | 3 (2.3%) | 1 (1.9%) | 1 (2.6%) | 0 (0.0%) | 0 (0.0%) |
| Superinfection | 0 (0.0%) | 1 (1.9%) | 0 (0.0%) | 1 (8.3%) | 0 (0.0%) |
| Thrombophlebitis | 0 (0.0%) | 1 (1.9%) | 0 (0.0%) | 1 (8.3%) | 0 (0.0%) |
| Tonsillitis | 8 (6.1%) | 2 (3.7%) | 1 (2.6%) | 0 (0.0%) | 1 (25.0%) |
| Typhoid fever | 1 (0.8%) | 7 (13.0%) | 3 (7.9%) | 4 (33.3%) | 0 (0.0%) |
| Unspecified infection | 1 (0.8%) | 0 (0.0%) | 0 (0.0%) | 0 (0.0%) | 0 (0.0%) |
| Urinary tract infection | 4 (3.1%) | 1 (1.9%) | 1 (2.6%) | 0 (0.0%) | 0 (0.0%) |

*OFI: other febrile illnesses*
