# Supplementary material for: C-reactive protein as a potential biomarker for disease progression in dengue: a multi-country observational study
Source: BMC Med. 2020 Feb 17;18:35. doi: 10.1186/s12916-020-1496-1 (PMC7025413; doi:10.1186/s12916-020-1496-1)
Supplement: Supplementary file 6 — Additional file 6: Figure S2. Association between CRP level and severe or intermediate dengue among dengue patients. [file 12916_2020_1496_MOESM6_ESM.docx]

**Additional file 6: Figure S2. Association between CRP level and severe or intermediate dengue among dengue patients**

**
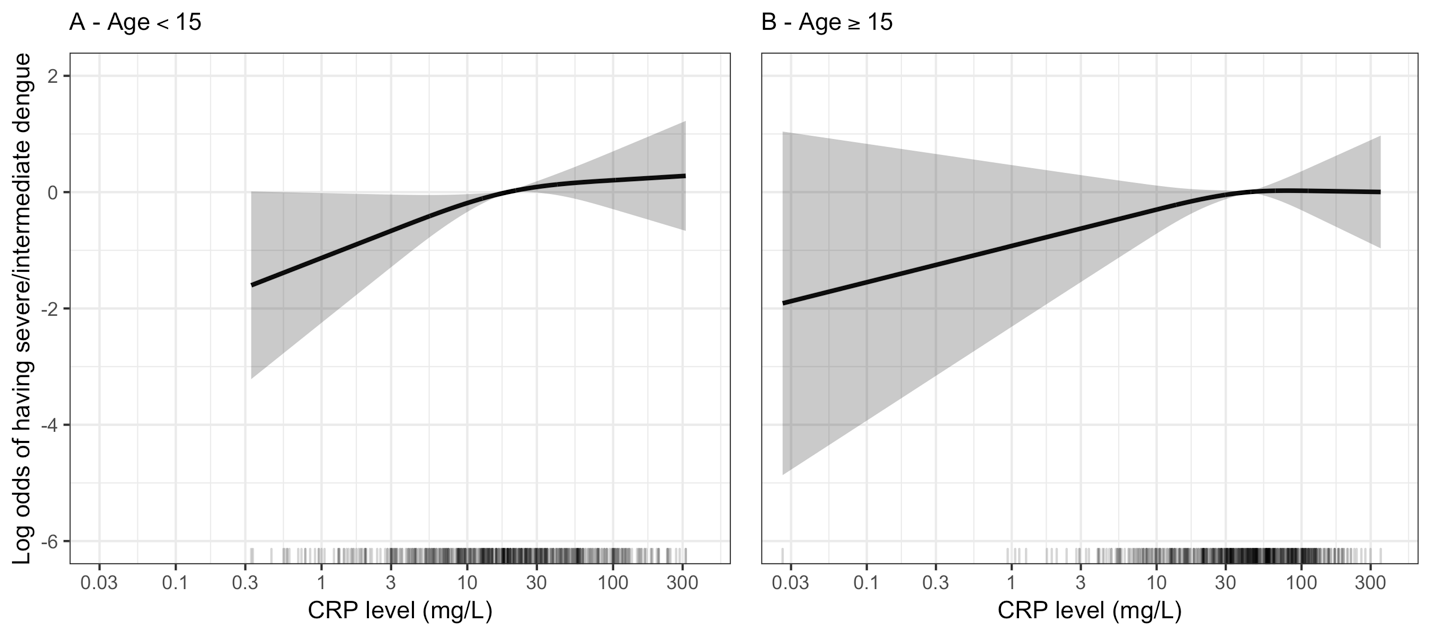
**

*The log odds of severe or intermediate dengue (the black line) and its 95% confidence interval (the grey region) were estimated from multivariable logistic regression models allowing for non-linear effect of log 2 of CRP levels using restricted cubic splines and adjusted for age, DOI at enrolment, viremia levels at enrolment, and immune status. P-values of the non-linear effect of CRP levels were 0.347 and 0.383 for A and B, respectively. The rug plot on the x-axis represents the distribution of individual cases.*

*CRP: C-reactive protein*
