# Supplementary material for: C-reactive protein as a potential biomarker for disease progression in dengue: a multi-country observational study
Source: BMC Med. 2020 Feb 17;18:35. doi: 10.1186/s12916-020-1496-1 (PMC7025413; doi:10.1186/s12916-020-1496-1)
Supplement: Supplementary file 9 — Additional file 9. Association between CRP level and clinical outcomes: sensitivity analysis including 9 cases with CRP values above the upper range of the assay. [file 12916_2020_1496_MOESM9_ESM.docx]

**Additional file 9: Association between CRP level and clinical outcomes: sensitivity analysis including 9 cases with CRP values above the upper range of the assay**

| Outcome | OR/HR | 95% CI | p-value |
| --- | --- | --- | --- |
| Severe or intermediate dengue^1^ | 1.17 | 1.06 – 1.28 | 0.001 |
| Severe dengue^1^ | 1.05 | 0.85 – 1.31 | 0.668 |
| Fever clearance time*^2^ |  |  |  |
| - CRP < 30 mg/L | 1.03 | 0.97 – 1.09 | 0.373 |
| - CRP ≥ 30 mg/L | 0.84 | 0.76 – 0.92 | <0.001 |
| Hospitalization^2^ |  |  |  |
| - CRP < 30 mg/L | 0.92 | 0.82 – 1.04 | 0.194 |
| - CRP ≥ 30 mg/L | 1.33 | 1.12 – 1.59 | 0.001 |

*The estimates (OR and HR) and 95% CI were reported for each one log 2 increase of CRP level, i.e. for each 2 times increase of CRP level*

**We use hazard ratio (HR) to report the results of Cox model for fever clearance time outcome. All other outcomes are reported by odds ratio (OR) estimated from logistic regression model. All models are multivariable models adjusted for age, DOI at enrolment, plasma viremia level, and immune status*

*^1^The model for severe or intermediate dengue was performed with linear effect of log 2 of CRP.*

*^2^The models for fever clearance time, hospitalization, and having dengue were performed with two separated linear effect of log 2 of CRP (CRP < 30 and CRP ≥ 30 mg/L).*

*CI: confidence interval; CRP: C-reactive protein; DOI: day of illness; HR: hazard ratio; OR: odds ratio*
