# Supplementary material for: C-reactive protein as a potential biomarker for disease progression in dengue: a multi-country observational study
Source: BMC Med. 2020 Feb 17;18:35. doi: 10.1186/s12916-020-1496-1 (PMC7025413; doi:10.1186/s12916-020-1496-1)
Supplement: Supplementary file 10 — Additional file 10: Figure S5. Association between CRP and other laboratory tests result. [file 12916_2020_1496_MOESM10_ESM.docx]

**Additional file 10: Figure S5. Association between CRP and other laboratory tests result**


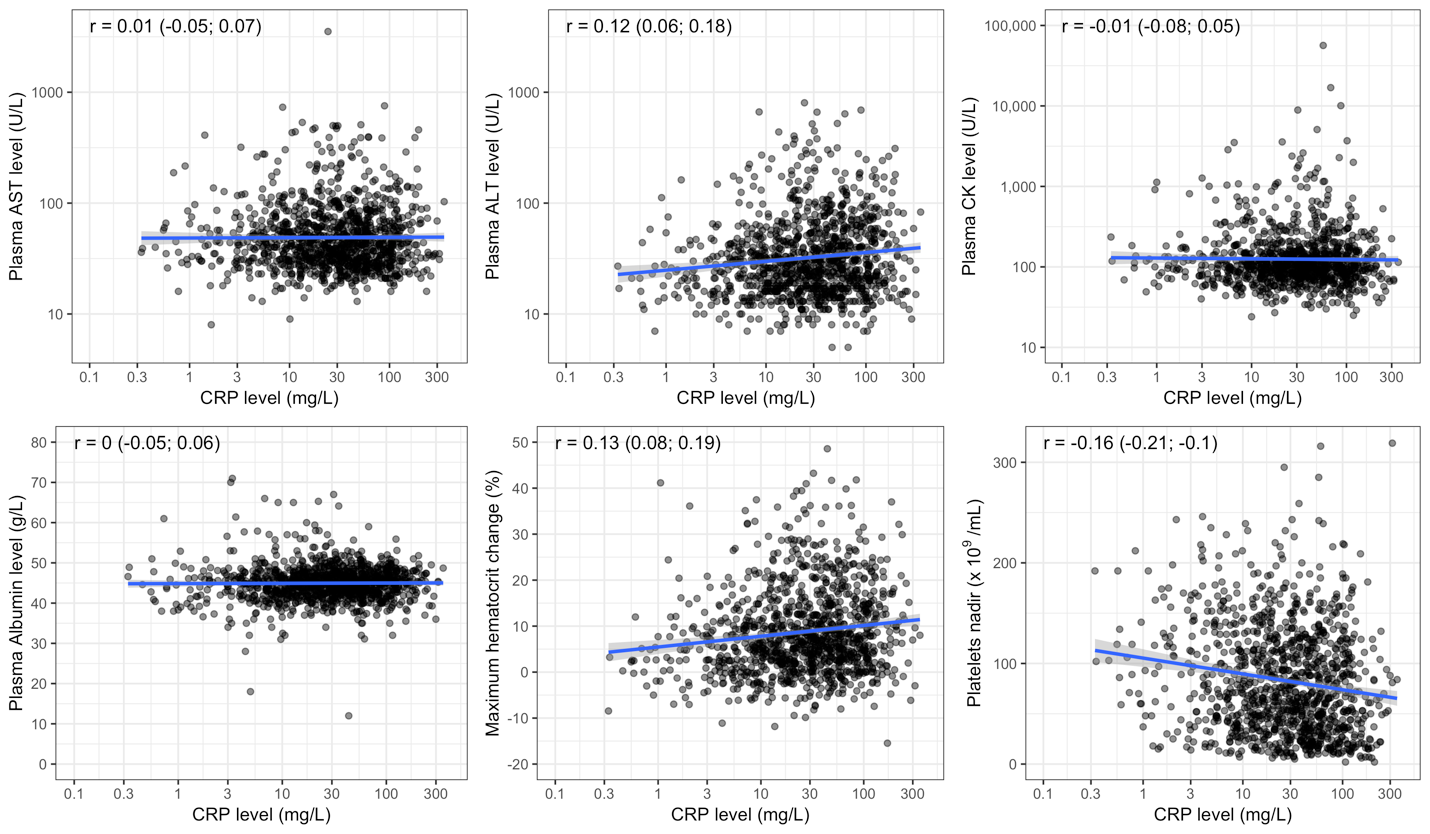


*The black point is the actual value for each patient. The blue line is the linear regression line and the grey region is its 95% confidence interval. Pearson’s correlation coefficient and its 95% confidence interval are shown in the top left corner of each plot. The x-axis is transformed using base-2 logarithm.*

*ALT: Alanine aminotransferase; AST: Aspartate aminotransferase; CK: Creatine kinase; CRP: C-reactive protein*
