# Supplementary material for: C-reactive protein as a potential biomarker for disease progression in dengue: a multi-country observational study
Source: BMC Med. 2020 Feb 17;18:35. doi: 10.1186/s12916-020-1496-1 (PMC7025413; doi:10.1186/s12916-020-1496-1)
Supplement: Supplementary file 11 — Additional file 11. Correlation between CRP and other biomarkers. [file 12916_2020_1496_MOESM11_ESM.docx]

**Additional file 11: Correlation between CRP and other biomarkers**

|  | All dengue patients | | Uncomplicated dengue patients | | Intermediate dengue patients | | Severe dengue patients | |
| --- | --- | --- | --- | --- | --- | --- | --- | --- |
| Biomarkers | Correlation coefficient | p | Correlation coefficient | p | Correlation coefficient | p | Correlation coefficient | p |
| Albumin | 0.005 (-0.054; 0.064) | 0.878 | -0.009 (-0.077; 0.059) | 0.791 | 0.123 (-0.004; 0.246) | 0.058 | 0.038 (-0.285; 0.353) | 0.822 |
| Lymphocytes | -0.363 (-0.413; -0.311) | <0.001 | -0.344 (-0.403; -0.283) | <0.001 | -0.359 (-0.465; -0.244) | <0.001 | -0.626 (-0.788; -0.383) | <0.001 |
| Neutrophils | 0.299 (0.244; 0.352) | <0.001 | 0.293 (0.230; 0.354) | <0.001 | 0.244 (0.121; 0.360) | <0.001 | 0.454 (0.157; 0.676) | 0.004 |
| WBC | 0.253 (0.197; 0.307) | <0.001 | 0.276 (0.212; 0.337) | <0.001 | 0.196 (0.071; 0.314) | 0.002 | 0.136 (-0.192; 0.437) | 0.415 |
| ALT | 0.118 (0.060; 0.176) | <0.001 | 0.137 (0.070; 0.203) | <0.001 | -0.025 (-0.150; 0.102) | 0.704 | 0.253 (-0.073; 0.53) | 0.126 |
| AST | 0.007 (-0.052; 0.066) | 0.817 | 0.014 (-0.054; 0.082) | 0.680 | -0.112 (-0.235; 0.015) | 0.083 | 0.015 (-0.306; 0.333) | 0.928 |
| CK | -0.014 (-0.076; 0.047) | 0.651 | 0.004 (-0.067; 0.075) | 0.906 | -0.088 (-0.217; 0.044) | 0.192 | -0.196 (-0.530; 0.191) | 0.317 |
| Maximum HCT change | 0.135 (0.077; 0.192) | <0.001 | 0.134 (0.067; 0.201) | <0.001 | 0.017 (-0.109; 0.143) | 0.792 | 0.017 (-0.309; 0.339) | 0.922 |
| PLT nadir | -0.157 (-0.214; -0.099) | <0.001 | -0.131 (-0.197; -0.064) | <0.001 | -0.118 (-0.240; 0.009) | 0.068 | -0.096 (-0.408; 0.235) | 0.571 |

*The Pearson’s correlation coefficients and p-values for these coefficients are reported by all dengue patients and each group of severity (uncomplicated, intermediate, and severe dengue). ALT: Alanine aminotransferase; AST: Aspartate aminotransferase; CK: Creatine kinase; CRP: C-reactive protein; HCT: haematocrit; PLT: platelet*
